# Application of a low-cost instrument to identify risk for hearing disorders in a municipal health network

**DOI:** 10.1590/2317-1782/e20250194en

**Published:** 2026-08-03

**Authors:** Monize Menon, Seisse Gabriela Gandolfi Sanches, Alessandra Giannella Samelli

**Affiliations:** 1 Departamento de Fisioterapia, Fonoaudiologia e Terapia Ocupacional, Faculdade de Medicina, Universidade de São Paulo – USP - São Paulo (SP), Brasil.

**Keywords:** Hearing, Mass Screening, Student, Diagnosis, Low-Cost Technology

## Abstract

**Purpose:**

To evaluate the performance of the PETIT application (automated tablet-based screening) compared with conventional sweep screening in identifying the risk of hearing impairment in school-aged children, using headphones with passive noise cancellation.

**Methods:**

Cross-sectional observational study including 122 children (aged 5 to 8 years) from the public school of Santana de Parnaíba. The following procedures were performed: otoscopy; automated hearing screening via tablet (with Sennheiser HD-280 headphones); and conventional sweep screening. Part of the sample also underwent pure-tone audiometry. Statistical analysis used binomial and binary logistic regression tests, along with the calculation of sensitivity, specificity, and accuracy.

**Results:**

There was significant association between the results of the sweep screening and PETIT, as well as the coefficient of the predictor variable (conventional audiometry) was significant to the performance in the sweep screening. For PETIT, a sensitivity of 85.7%, specificity of 100%, and accuracy of 99.18% were observed.

**Conclusion:**

The results suggest that this tool has promising applicability in schools for identifying children at risk of hearing impairments.

## INTRODUCTION

Several studies acknowledge that hearing loss affects language development and academic performance in children when not detected early, generating negative impacts on quality of life^(1-4)^. The World Health Organization (WHO) estimates that more than 80% of people with some degree of hearing loss reside in low- and middle-income countries, where access to hearing health services is limited or non-existent^(4)^.

The Joint Committee on Infant Hearing^(5)^ recommended early identification, diagnosis, and intervention in hearing loss, proposing universal hearing screening for all newborns. However, hearing assessment programs should not be limited to the neonatal period, since hearing loss may manifest later in some cases. Thus, monitoring and assessing hearing should be a continuous process throughout the different stages of development^(6)^.

Otitis media, one of the main causes of hearing loss in childhood, has a global annual incidence of approximately 10.85%, representing more than 700 million new cases per year. This rate can vary between regions and countries, ranging from 3.6% in Central Europe to over 43% in African countries, due to genetic predispositions and modifiable risk factors, such as allergies, upper respiratory tract infections, exposure to tobacco smoke, inadequate sanitation, malnutrition, and low socioeconomic status^(4)^.

In Brazil, data from the National Health Survey (PNS-2019) indicate that approximately 1.1% of the population aged 2 years or older reported "extreme difficulty or inability to hear," corresponding to approximately 2.3 million Brazilians^(7)^. Previous studies with Brazilian schoolchildren found a prevalence of hearing loss varying between 5% and 16%, depending on the age group assessed and the screening method used^(8,9)^.

Brazil has a Hearing Healthcare Network structured within the Unified Health System (SUS) since Ordinance No. 2,073/2004, updated and incorporated into the National Policy for the Healthcare of People with Disabilities. This network establishes integrated flows for screening, diagnosis, provision of hearing aids, and rehabilitation, with regional referral centers and services qualified at different levels of complexity. Although there are gaps related to coverage, equity, and continuity of care, Brazil has one of the most organized public hearing health networks in Latin America, with progressive expansion since the creation of the National Hearing Healthcare Program^(9,10)^.

The School Health Program (PSE), established by Decree No. 6,286/2007, aims to integrate actions for promotion, prevention, and hearing screening at school, expanding access for children and strengthening the articulation between the health and education sectors. The PSE has contributed to the early identification of hearing impairments in schoolchildren, promoting continuity of care in municipal education networks^(11,12)^. Thus, Brazil has a structured, integrated, and articulated model of hearing care, with intersectoral policies, guaranteeing access to specialized services and prevention and screening strategies at different levels of healthcare. Nevertheless, there are limitations and regional inequalities^(11-13)^.

It is important to highlight that the incidence of late-onset hearing loss during school age can increase by 16.4%, with mild hearing loss being more frequent than moderate or profound hearing loss^(14-16)^.

The WHO emphasizes that early and lower-cost interventions, such as hearing screening, can significantly reduce the social, educational, and economic impact of unidentified hearing loss, especially in school populations^(4,16)^.

Hearing loss is diagnosed through conventional audiological evaluation, especially pure-tone audiometry, which is the gold standard and requires a clinical audiometer^(17)^. This may hinder access to evaluation, since the audiometer can be more expensive and depends on a qualified professional and a quiet place to perform it^(4,17)^, making access to this service difficult in many locations.

With the advancement of technology, some tools have been evaluated to facilitate the identification of risk for hearing impairment. The results have favored their use at school to expand hearing healthcare, proving valid for this purpose^(18,19)^. However, the noise in these environments can impair hearing screenings, providing false-positive results. To minimize the impact of noise on assessments in environments without acoustic treatment, a previous study recommended the use of headphones with passive noise reduction^(20)^.

Thus, given the difficulty encountered regarding the scarcity of resources for the early identification of hearing impairments in the public health network, especially in low- and middle-income countries, this study aims to contribute to the improvement of new, lower-cost automated hearing screening method performed at school, including it as part of the PSE, in a municipality in the State of São Paulo, Brazil.

Thus, this study aimed to evaluate the performance of the PETIT application (automated screening on a tablet), compared to audiometric sweep screening, in identifying the risk of hearing impairment in schoolchildren, using headphones with passive noise cancellation.

## METHODS

This study was approved by the institution's Ethics Committee, under number CAEE 07377319.8.0000.0065. All guardians of the children participating in this study signed an informed consent form, and the children signed an assent form.

### Sample

The sample for this study was constituted by non-probabilistic convenience sampling, comprising children enrolled in municipal schools, selected according to their availability to participate during the data collection period and according to the inclusion criteria. The choice of convenience sampling is justified by the care context in which the research was carried out and by the feasibility of accessing the participants at school.

The study included 122 children enrolled in the 1^st^ and 2^nd^ grades in municipal schools of the city of Santana de Parnaíba, Brazil. The inclusion criteria were an age range from 5 years to 8 years and 11 months; signature of an informed consent form by the guardians and assent form by the children; otoscopy without indication of foreign bodies or excess cerumen; understanding, on the part of the child, of the instructions for performing the screening by tablet, audiometric sweep screening, and pure-tone audiometry.

### Materials and equipment

The study used an Apple iPad tablet with the PETIT^(11)^ and NoiSee applications; HD 280 PRO headphones connected to the iPad; MAICO MA42 audiometer; TDH39 headphones; Heine mini 3000 otoscope; and a computer for data recording.

### Procedures

**Figure 1:**
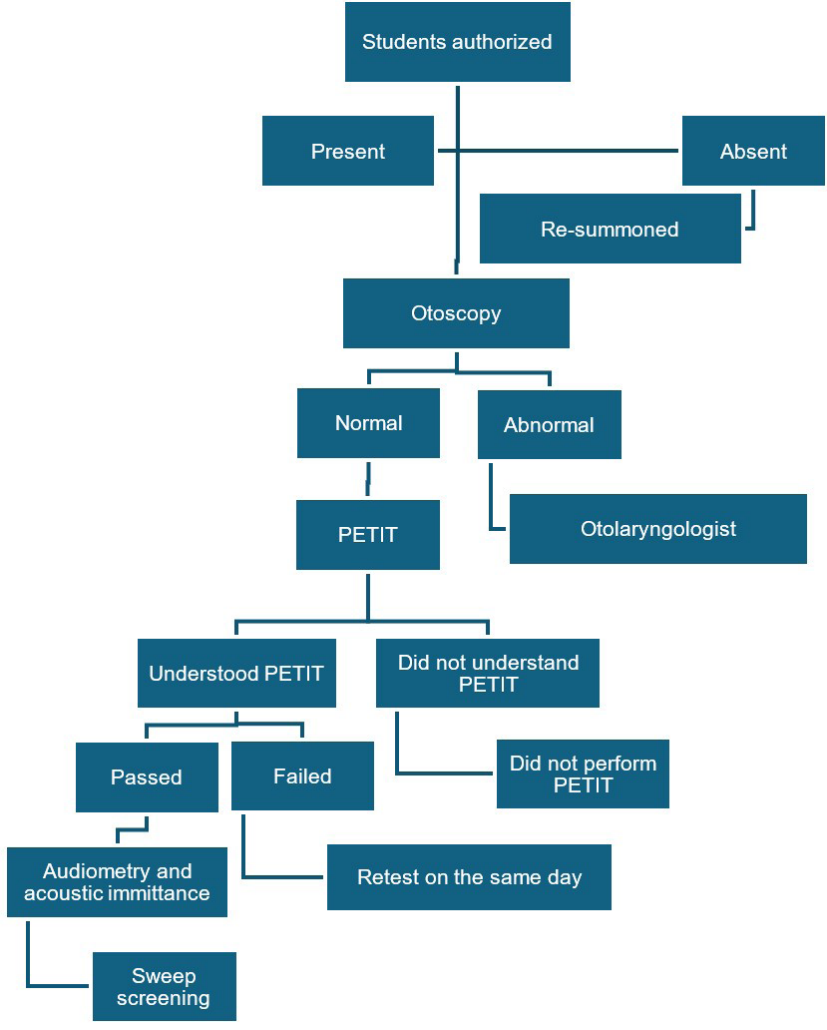
Flowchart of the study procedures (Santana de Parnaíba, SP, 2025)

The study was conducted in stages: parental authorization, hearing screening with PETIT, complete audiological evaluation (otoscopy, pure-tone audiometry, speech audiometry, and acoustic immittance measurements), and audiometric sweep screening, as described below. The study stages are shown in the flowchart (Figure 1).

#### Hearing screening - PETIT

Subjects underwent hearing screening at their school during the school year. Initially, a visual inspection of the auditory canal was performed, followed by hearing screening.

The hearing screening was performed using the HD 280 PRO headset, through the application installed on a tablet: Hearing Screening Extension Program by Tone – PETIT^(18)^, properly calibrated. The screening is conducted through an interactive game with pure tones (warble), using a combination of visual and auditory stimuli, with a forced-choice response paradigm (yes or no), tested and validated in schoolchildren using TDH-49 headphones^(18)^. The application uses an algorithm that evaluates frequencies of 1000, 2000, and 4000 Hz at 20 dBHL and 500 Hz at 30 dBHL, in each ear separately. For the individual to pass the screening, responses to at least two of the three emitted tones are required^(21,22)^: 20 dBHL for 1000, 2000, and 4000 Hz and 30 dBHL for 500 Hz, in both ears. At the end of the screening, the application provides the result as "Passed" or "Failed," which is stored on the tablet and sent to the central database via Wi-Fi for later analysis^(18)^. The latter considered the results with and without the 500 Hz frequency (using an analysis filter, which is part of the application). This analysis was performed as a methodological control, due to the greater susceptibility to errors at this frequency caused by noise interference at school^(18)^.

#### Complete audiological evaluation

Following the screenings, all children were referred for a complete audiological evaluation at the nearest audiology referral center in Santana de Parnaíba by its speech-language-hearing pathologists. Among the 88 (66.7%) participants who scheduled the complete audiological evaluation, 45 (51.1%) attended the specialized service, and 43 (48.9%) did not attend. Of the 45 exams performed, 41 (91.1%) were received by the researcher, but only 39 were complete, containing all the records required for analysis (hearing thresholds at all tested frequencies, tympanometry curves, and acoustic reflexes). Thus, due to the low adherence to the complete audiological evaluation, a new strategy was adopted for comparison with the PETIT results, namely, Audiometric sweep screening.

#### Audiometric sweep screening

Audiometric sweep screening was also applied at school, during the school year, on a different day from the screening with PETIT, using a portable audiometer model MA42 (MAICO), properly calibrated according to international safety standards, and with the use of TDH39 headphones.

The sweep screening followed the protocol, with presentation of warble pure tones at 1000, 2000, and 4000 Hz at 20 dBHL and 500 Hz at 30 dBHL^(21,22)^. The approval criterion followed internationally standardized guidelines: to “pass” in the hearing screening, the participant had to respond correctly to at least two of the three tones at 1000, 2000, and 4000 Hz and to the stimulus at 500 Hz, in each ear^(21,22)^. At the end of each screening, the researcher recorded the results manually in a standardized physical protocol, with each participant individually classified as “Passed” or “Failed”, according to their performance^(23)^.

### Data analysis

The results obtained in the PETIT screening were compared to the sweep screening test to obtain the diagnostic hearing screening values: sensitivity, specificity, and accuracy. The complete audiological evaluation was compared with the audiometric sweep screening test to establish the validity of the sweep screening test as a procedure equivalent to the gold standard (pure-tone audiometry) for the analyses. Binary logistic regression models were applied to evaluate the association between the methods (Model 1: Sweep screening test as a predictor of the PETIT result; Model 2: Pure-tone audiometry as a predictor of the sweep screening test result). The goodness of fit of the models was analyzed based on Deviance; AIC (Akaike Information Criterion); BIC (Bayesian Information Criterion); Pseudo-R^2^: McFadden, Cox & Snell, Nagelkerke, and Tjur. The significance of the model coefficients was evaluated using the Wald test, considering a 5% significance level. Diagnostic performance measures (sensitivity, specificity, and accuracy) were calculated based on the confusion matrix generated in each model. The binomial test was used to compare the observed pass rates in the hearing screening. The p-values, in this case, refer to the observed rate with the reference value of 0.5 (50%). Analyses were performed using JASP software, version 0.19.3.0. Results were considered statistically significant when p < 0.05.

## RESULTS

The sample consisted of 122 children, aged 5 to 8 years, with a median age of 7 years and a mean age of 6.75 years (± 0.64). There was no significant difference between the sexes (42.6% female; 57.4% male; p = 0.123; Binomial test).

The chi-square test of independence with continuity correction was applied to verify the existence of an association between the participants' performance on the PETIT test and sex. Among seven individuals who failed the screening, three were female, and four were male, with no significant difference (chi-square with continuity correction: Χ^2^ = 2.608×10^(−30)^; df = 1; p = 1.000).

Table 1 presents the descriptive analysis of the sweep screening by frequency tested in both ears, the overall results by ear, and the final classification of passed (P) or failed (F). The overall results by ear show that five participants failed at least one frequency in the right ear (4.1%) and three in the left ear (2.5%). In the end, six children (4.9%) were classified as having failed the sweep screening, while 116 (95.1%) passed. All these proportions were significantly different from the reference value of 50% (p < 0.001), evidencing an adequate overall auditory performance in the sample evaluated.

**Table 1:** Results of audiometric sweep screening by frequency and ear in public school students (Santana de Parnaíba, SP, 2025)

**Variable**	**Result**	**Frequency**	**Total**	**Proportion**	**P**
RE 500 Hz	F	1	122	0.008	< .001
	P	121	122	0.992	< .001
RE 1000 Hz	F	2	122	0.016	< .001
	P	120	122	0.984	< .001
RE 2000 Hz	F	1	122	0.008	< .001
	P	121	122	0.992	< .001
RE 4000 Hz	F	3	122	0.025	< .001
	P	119	122	0.975	< .001
LE 500 Hz	F	0	122	0.000	< .001
	P	122	122	1.000	< .001
LE 1000 Hz	F	3	122	0.025	< .001
	P	119	122	0.975	< .001
LE 2000 Hz	F	2	122	0.016	< .001
	P	120	122	0.984	< .001
LE 4000 Hz	F	2	122	0.016	< .001
	P	120	122	0.984	< .001
RE (overall)	F	5	122	0.041	< .001
	P	117	122	0.959	< .001
LE (overall)	F	3	122	0.025	< .001
	P	119	122	0.975	< .001
Passed/Failed	Failed	116	122	0.951	< .001
	Passed	6	122	0.049	< .001

Binomial test with a reference value of 0.5

**Caption:** RE= right ear, LE= left ear, F = failed; P = passed

The results indicate that the vast majority of the children evaluated passed the PETIT test, regardless of the ear or condition analyzed. Specifically, 117 children (95.9%) passed in the left ear, and 116 (95.1%) passed in the right ear. An analysis was also performed, excluding the 500 Hz frequency, to verify a possible influence of noise on the participants' performance. However, the pass rates remained identical for the analyses including and excluding 500 Hz, with 95.9% passing in the left ear and 95.1% in the right ear (Table 2). All results were statistically significant (p < 0.001) when compared to the reference value of 50%, indicating that the pass rates are significantly higher than chance. The overall PETIT classification shows that 115 children (94.3%) passed the hearing screening, while seven (5.7%) failed.

**Table 2:** Results of automated hearing screening with the PETIT application, with and without the 500 Hz frequency, in public school students (Santana de Parnaíba, SP, 2025)

**Variable**	**Result**	**Frequency (n)**	**Total**	**Proportion**	**p**
PETIT LE	P	117	122	0.959	< .001
	F	5	122	0.041	< .001
PETIT RE	P	116	122	0.951	< .001
	F	6	122	0.049	< .001
PETIT LE (-500 Hz)	P	117	122	0.959	< .001
	F	5	122	0.041	< .001
PETIT RE (-500 Hz)	P	116	122	0.951	< .001
	F	6	122	0.049	< .001
PETIT Passed/Failed	Passed	115	122	0.943	< .001
	Failed	7	122	0.057	< .001

The p-values ​​refer to the binomial test, comparing the observed proportion with the reference value of 0.5

**Caption:** LE = left ear; RE = right ear; P = passed; F = failed

A binary logistic regression analysis was performed to investigate the relationship between participants' performance on the PETIT and the results of the sweep screening. The objective was to verify if the sweep screening result (predictor variable) could significantly explain the scores obtained on the PETIT (dependent variable). Table 3 presents the fit indicators of the two models tested: the null model (M_0_), which does not include predictor variables, and the complete model (M_1_), which includes the sweep screening result as a predictor. Model M_1_ presented a significantly lower chi-square value than the null model (Χ^2^(120) = 42.105; p < 0.001), indicating that the inclusion of the sweep screening results substantially improved the predictive capacity of the model. The McFadden R^2^ value was 0.785, considered high for logistic models, which reinforces the quality of the fit and the explanatory power of the predictor variable on the PETIT scores. This value indicates that the model explains approximately 78.5% of the variance in the PETIT results, which is highly satisfactory for this type of analysis. The estimated coefficients and their respective Wald tests are also presented in Table 3. Although the intercept of the M_1_ model was statistically significant (p < 0.001), the coefficient associated with PASSED/FAILED in the sweep screening showed a statistically non-significant result (p = 0.996), with an extremely high standard error. This pattern may indicate collinearity or a near-perfect fit between the variables, which tends to inflate the standard errors in logistic models.

**Table 3:** Binary logistic regression model for analyzing the performance of PETIT in relation to audiometric sweep screening (Santana de Parnaíba, SP, 2025)

**Model**	**Deviance**	**AIC**	**BIC**	**df**	**ΔΧ^2^**	**p**		**R^2^ McFadden**	**R^2^ Nagelkerke**	**R^2^ Tjur**	**R^2^ Cox & Snell**	
M_0_	53.604	55.604	58.408	121	—	—		—	—	—	—	
M_1_	11.499	15.499	21.107	120	42.105	< .001		0.785	0.821	0.850	0.292	
**Coefficients of the M_1_ model of logistic regression**												
**Variable**			**Estimate**		**Standard error**		**Z**		**Wald Statistics**	**df**		**p**
Intercept (M_1_)			−4.745		1.004		−4.724		22.320	1		< .001
Sweep screening Passed/Failed			24.311		4390.308		0.006		3.066 × 10^(−5)^	1		0.996

**Caption:** M_1_ includes Sweep screening Passed/Failed

The performance metrics (Table 4) indicate a total accuracy of 99.2%, demonstrating the model's high ability to correctly classify cases based on sweep screening results. The sensitivity was 85.7%, representing the proportion of cases with hearing impairment (failure) that were correctly identified by the model. The specificity was 100%, indicating that all cases with hearing within the normal range were correctly classified as such.

**Table 4:** Performance of PETIT compared to audiometric sweep screening (Santana de Parnaíba, SP, 2025)

**Observed/Predicted**	**Passed**	**Failed**	**% Correct**
Passed	115	0	100.0%
Failed	1	6	85.7%
**Overall total**			**99.2%**
**Metrics**			**Value**
Accuracy			0.992
Sensitivity			0.857
Specificity			1.000

Moreover, a binary logistic regression was conducted to verify the validity of the sweep screening as a substitute technique for pure-tone audiometry (considered the gold standard), using sweep screening results as the dependent variable and audiometry results as the predictor variable. This analysis was performed complementarily to validate the use of the sweep screening as a comparative technique in this study. Thus, this analysis serves as methodological support, reinforcing the legitimacy of the use of the sweep screening in the comparative tests performed. The applied logistic regression revealed that the model with a predictor (M_1_) performed better than the null model (M_0_), with a reduction in the values ​​of Deviance, AIC, and BIC, as shown in Table 5A. The chi-square difference between the models was ΔΧ^2^(1) = 3.810, with a p-value of 0.051, indicating a trend towards statistical significance. Although the p-value is slightly above the adopted significance level (0.05), the pseudo-R^2^ coefficients, especially McFadden's coefficient (0.241), indicate a satisfactory fit of the model, suggesting that the sweep screening is significantly associated with the audiometry result. Table 5A also presents the estimated regression coefficients.

**Table 5:** Binary logistic regression model for analyzing the performance of sweep screening in relation to pure-tone audiometry (gold standard) (A). Performance of audiometric sweep screening compared to pure-tone audiometry (gold standard) (B) (Santana de Parnaíba, SP, 2025)

**A – Logistic regression models**														
**Model**	**Deviance**		**AIC**	**BIC**		**Df**	**ΔΧ^2^**	***p***		**R^2^ McFadden**	**R^2^ Nagelkerke**		**R^2^ Tjur**	**R^2^ Cox & Snell**
M_0_	15.777		17.777	19.441		38	—	—		—	—		—	—
M_1_	11.967		15.967	19.294		37	3.810	0.051		0.241	0.280		0.224	0.093
**Coefficients of the logistic regression model**														
**Model**		**Term**			**Estimate (β)**			**Standard error**		***Z***	**Wald χ^2^**		***df***	***p***
M_0_		Intercept			-2.918			0.726		-4.019	16.155		1	<0.001
M_1_		Intercept			0.000			1.414		≈0	≈0		1	1.000
M_1_		Audiometry (0)			-3.584			1.740		-2.059	4.241		1	0.039
**B – Performance**														
**Observed/Predicted**									**Passed (0)**			**Failed (1)**		**% Correct**
Normal									36			1		97.3%
Abnormal									1			1		50.0%
**Total**														**94.9%**
**Metrics**														**Value**
Accuracy														0.949
Sensitivity														0.500
Specificity														0.973

The M_1_ model includes the audiometry result as a predictor variable. The category “1” represents failure (abnormal). The variable “audiometry” was coded with “1” for failure and “0” for normality

The coefficient of the predictor variable (audiometry) was statistically significant (p = 0.039), indicating that the audiometry result is a relevant predictive factor for the child's performance on the screening test. The odds ratio, derived from the exponentiation of the coefficient, would indicate a sharp drop in the chances of passing the screening test when there is a failure in audiometry, which reinforces the validity of the screening test as a substitute method for audiometry. Table 5B presents the model's performance, which showed an overall accuracy of 94.9%, with only two classification errors (one false positive and one false negative). Specificity was high (97.3%), indicating a high capacity of the model to correctly recognize normal cases. On the other hand, sensitivity was 50%, reflecting a moderate capacity to detect altered cases. Finally, the pseudo-R^2^ values​(McFadden = 0.241; Nagelkerke = 0.280; Tjur = 0.224) are within the expected parameters for well-fitted logistic models, which corroborates the quality of the fit and the usefulness of the sweep screening in this context.

## DISCUSSION

The participants’ auditory performance and sex results in the PETIT screening indicate no significant difference between the sexes, corroborating the idea that the children’s auditory performance was not influenced by this variable. This finding is consistent with previous studies, which did not observe a difference in performance between boys and girls in similar auditory assessments^(19,24,25)^. Such evidence suggests that, when considering auditory screening, there is no need for adjustments or different approaches based on sex, which facilitates the universal application of these procedures.

Regarding audiometric sweep screening, the data showed that most participants had auditory function within the expected standards for their age range, with reduced failure rates. The overall pass rate in the procedure was 95.1%, with only six participants (4.9%) being classified as having failed the sweep screening in at least one of the frequencies tested.

Previous studies demonstrate variability in the "pass/fail" classification rate in infant hearing screenings, depending on factors such as the child's age, the application environment, the criteria adopted for failure, and the methodology employed^(25-27)^. Higher failure rates are commonly found in school settings, for example, due to less controlled acoustic conditions, less cooperation from some participants, or transient factors, such as cerumen or upper respiratory tract infections^(14,15,28,29)^.

The heterogeneity in failure rates in school hearing screenings can be attributed to several factors, including differences in screening protocols, reference criteria, environmental conditions during testing, and demographic characteristics of the populations evaluated. Studies conducted in different geographic and socioeconomic contexts demonstrate significant variations in failure rates, even when employing similar methods, such as pure-tone audiometry^(30)^. A study conducted in Poland with 67,416 schoolchildren reported a failure rate of 16.4% in hearing screening^(28)^ among children aged 6 to 13 years. A study conducted in Saudi Arabia^(31)^ with 308 first-grade children found a screening failure rate of 26.3% using pure-tone audiometry. Thus, the failure rate of 4.9% observed in the present study is lower than that observed in previous studies and may reflect the aforementioned methodological differences.

Only seven participants (5.7%) failed the PETIT, indicating a low screening failure rate. Despite the low prevalence of failures in this study, it is important to systematically implement protocols such as PETIT in childhood hearing screening programs. Cases identified as suspected of hearing loss should be referred for complete audiological evaluation, as recommended by national^(32)^ and international^(5)^ guidelines, due to the importance of early diagnosis of hearing impairments to avoid or mitigate harm to child development.

Regarding the predictive validity of the PETIT compared to the sweep screening, the findings demonstrated high agreement between the instruments, as they indicated that the sweep screening results are strongly associated with the PETIT results, with approximately 80% of the variance in the PETIT results being explained by the sweep screening. This suggests that the PETIT can be considered a valid instrument for childhood hearing screening.

From the performance standpoint, the accuracy of PETIT with HD 280 headphones was 99.2%, specificity was 100%, and sensitivity was 85.7%. This means that the PETIT with headphones with passive noise suppression was able to correctly identify all individuals who passed the sweep screening (true negatives) and most (~86%) of the cases that failed (true positives), showing high accuracy – i.e., there was a correct classification of "normal" and "abnormal" cases for the vast majority of those evaluated (99.2%). A previous study^(18)^ identified that performance metrics varied among the different hearing screening methods evaluated: sensitivities ranged from 60% to 95%, specificities from 44% to 91%, and accuracy values ​​from 49% to 88%; the best results were obtained with the tablet-based screening method and the serial approach. Another study^(33)^ with schoolchildren of the same age range as the present study, which used an automated hearing screening with TDH headphones compared to audiometry (gold standard), observed an accuracy of 85%, sensitivity of 96%, and specificity of 81%. Thus, the performance obtained for the PETIT with headphones with passive noise suppression in this study was similar to that obtained in previous studies^(18,33)^.

The validation of screening instruments is a fundamental step to ensure their applicability in clinical and educational contexts. Because of its ease of application and behavioral nature, PETIT proves to be a viable alternative for contexts with limitations in technological or human resources, such as schools and community health centers, as per previous studies^(18,19)^. The results verified in the present study (which used HD 280 PRO headphones, while previous studies used TDH39 headphones) suggest reliability in the auditory screening performed through PETIT, indicating that this can be a useful tool in the early identification of risk for hearing impairments, and can minimize the negative impacts on children’s language development and school performance, as suggested by contemporary studies^(4,25)^.

To validate the use of the audiometric sweep screening test as a technique equivalent to the gold standard, we compared the results of the sweep screening test with pure-tone audiometry for those children who underwent audiometry and received a complete evaluation (n = 39). The model for this analysis showed an accuracy of 95%, specificity of 97%, and sensitivity of 50%. It is important to consider, especially given the observed sensitivity, that just over 30% of the children included in the previous analyses (PETIT and sweep screening test) also underwent conventional audiometry. Therefore, this low sensitivity is possibly related to the low adherence to audiometry and the low prevalence of hearing impairments detected in this sample; only two cases with abnormalities in the sweep screening test underwent audiometry, and in one of them, there was no correspondence between the results. This sampling bias is a recognized limitation in studies with an asymmetrical distribution of events, directly impacting the accuracy of measures such as sensitivity and logistic coefficients^(25,30)^. In addition, sensitivity can be impacted by factors such as cutoff criteria and environmental conditions^(25,30)^.

The high specificity verified in this analysis is a desirable characteristic for population screening tests, as it minimizes false positives, optimizing the referral of only suspected cases for complete diagnostic evaluation, avoiding the overload of specialized services^(4,25,30)^. However, when we consider the 95% accuracy between sweep screening and pure-tone audiometry, we can suggest that sweep screening presents itself as a viable tool for this purpose.

In this context and aligned with international recommendations^(4)^, hearing screening strategies that combine diagnostic accuracy with ease of application and low cost are fundamental to address the high prevalence of hearing loss, especially in vulnerable populations and in low- and middle-income countries, where the coverage of specialized audiological services is limited^(34-36)^. The systematic review conducted by Melo et al.^(30)^ reinforces that accessible methods with good diagnostic performance are fundamental for the early detection of hearing loss.

Although not the subject of this study, it is important to highlight that other variables should be considered during hearing screening programs in schoolchildren. These include prior contact with the school, previously surveying their medical history with family members and teachers to obtain development and learning data, recording environmental and behavioral factors that may interfere with the results, and so on^(1-4,37,38)^.

When discussing the performance of hearing screening in schoolchildren, it is also essential to analyze the cost-benefit of the program, including logistical and human factors involved in the process. A WHO report on hearing screening presented an assessment of the costs and financial returns associated with the implementation of hearing screening strategies and early intervention services in different contexts. According to the document, the global expansion of hearing screening coverage and intervention programs over the next 10 years would require an additional annual per capita investment of approximately US$1.33, resulting in significant public health gains, with the estimated prevention of about 130 million disability-adjusted life years, direct benefit to 1.4 billion individuals, and an average economic return of US$16 for every dollar invested. These data highlight the relevance of hearing screening, emphasizing the need for expansion and strengthening within national child and school hearing health programs^(37)^.

In the Brazilian context, the cost-benefit analysis of hearing screenings must also consider the structural barriers that can compromise the continuity of care, such as the waiting time for diagnostic evaluation, the scarcity of specialized professionals in certain regions, and the need for families to travel to referral services. A nationwide study found that the average waiting time for specialized care in hearing health referral services can exceed 6 months, especially in municipalities that do not have centers accredited by the SUS^(12)^.

The results of this study corroborate and expand on the findings of previous research^(38)^, which evaluated the performance of the PETIT with the HD 280 PRO headphones under controlled listening conditions, using white noise and cafeteria noise as simulations of noisy environments, in adults. In that study, the authors observed 100% sensitivity and specificity when the HD 280 PRO was used in conjunction with the PETIT, achieving 100% accuracy in both noise scenarios evaluated and superior performance to that of the TDH headphones. The present study applied the same tool in a real-world environment and to the target population, achieving an accuracy of 99.2%. This finding reinforces the hypothesis that the HD 280 PRO is a technically viable alternative to the TDH-39 headset, traditionally used in clinical audiometry, especially in contexts where hearing screening is performed in uncontrolled environments, such as schools. Even with potential sources of environmental noise typical of schools, the HD 280 PRO performed similarly to what was observed in the laboratory. The comparison between the studies suggests that the diagnostic performance verified in this study is compatible with that reported by Aquino^(38)^.

Furthermore, the HD 280 PRO headset costs less than the TDH-39 headset and features passive noise attenuation technology, which makes it particularly suitable for applications at school, where acoustic control is limited. The estimated cost of the HD 280 PRO is approximately US$89^(39)^, while that of the TDH-39 is around US$500^(40)^.

Thus, our results suggest that the protocol evaluated here is a viable tool for early identification of hearing impairments in schoolchildren, consistent with national and international literature. This validates the use of instruments such as PETIT in schools and clinics due to its sensitivity in detecting minimal hearing impairments^(2,11,12,18)^.

Using headphones with passive noise cancellation in conjunction with PETIT proved advantageous for its ease of handling and low cost, which expands its possibilities of use in remote, vulnerable regions or those with few available technical and human resources. Although the TDH-39 headphones are widely used in hearing screenings, their costs and logistical limitations may make their large-scale application in the educational context unfeasible. Thus, PETIT with lower-cost headphones is a viable alternative, with the potential to integrate public policies aimed at promoting hearing health.

Despite the promising results, some gaps remain and require further investigation. First, it is necessary to expand the sample diversity, including different age groups, geographic regions, and school contexts to enable the generalization of the findings. There is also a need for new studies investigating the use of mobile devices in hearing screening and the cost-benefit analysis of the implementation of low-cost instruments for hearing screening at schools.

## CONCLUSION

Our results indicate that hearing screening via tablet using headphones with passive noise cancellation obtained accuracy values ​​of 99.2%, sensitivity of 85.7%, and specificity of 100%, reinforcing its validity as a screening tool. Thus, hearing screening performed with lower-cost headphones proved to be a promising tool for school environments, especially in contexts with limited infrastructure.
