# Supplementary material for: Application of a low-cost instrument to identify risk for hearing disorders in a municipal health network
Source: Codas. 2026 Aug 3;38(5):e20250194. doi: 10.1590/2317-1782/e20250194en (PMC13445992; doi:10.1590/2317-1782/e20250194en)
Supplement: Translation

# Translation: Aplicação de instrumento de baixo custo na identificação de risco para alterações auditivas em uma rede municipal de saúde

**DOI:** 10.1590/2317-1782/e20250194pt

**Authors:** Monize Menon, Seisse Gabriela Gandolfi Sanches, Alessandra Giannella Samelli

**Affiliations:** 1 Departamento de Fisioterapia, Fonoaudiologia e Terapia Ocupacional, Faculdade de Medicina, Universidade de São Paulo – USP - São Paulo (SP), Brasil.

**Keywords:** Audição, Triagem, Estudantes, Diagnóstico, Tecnologia de Baixo Custo

## Abstract

**Objetivo:**

Avaliar o desempenho do aplicativo PETIT (triagem automatizada em tablet), em comparação com a varredura audiométrica, na identificação de risco para alterações auditivas em escolares, utilizando fones de ouvido com cancelamento de ruído passivo.

**Método:**

Estudo observacional transversal com 122 crianças (05 a 08 anos) da rede pública municipal de Santana de Parnaíba. Foram realizados meatoscopia; triagem automatizada via tablet (com fones Sennheiser HD-280); triagem por varredura audiométrica. Parte da amostra realizou também audiometria convencional (padrão-ouro). Para análise, foram utilizados: testes binomial e de regressão logística binária, assim como cálculo de sensibilidade, especificidade e acurácia.

**Resultados:**

Houve associação significativa entre os resultados da triagem por varredura e do PETIT, assim como o coeficiente da variável preditora (audiometria convencional) foi estatisticamente significativo ao desempenho na triagem por varredura. Para o PETIT, verificou-se sensibilidade de 85,7%, especificidade de 100% e 99,18% de acurácia.

**Conclusão:**

Os resultados sugerem que esta ferramenta tem aplicabilidade promissora em ambiente escolar, podendo facilitar a identificação de crianças de risco para alterações auditivas.

## INTRODUÇÃO

Diversos estudos reconhecem que a perda auditiva afeta o desenvolvimento de linguagem e desempenho acadêmico de crianças quando não detectada precocemente, gerando impactos negativos para a qualidade de vida^(1-4).^ A Organização Mundial da Saúde (OMS) estima que mais de 80% das pessoas com algum grau de perda auditiva residem em países de baixa e média renda, onde o acesso aos serviços de saúde auditiva é limitado ou inexistente^(4)^.

O Joint Committee on Infant Hearing^(5)^ recomendou a identificação, diagnóstico e intervenção precoce nas perdas auditivas, propondo a triagem auditiva universal, para todos os neonatos. Entretanto, os programas de avaliação auditiva não devem se limitar ao período neonatal, uma vez que, em alguns casos, a perda auditiva pode manifestar-se posteriormente. Assim, o acompanhamento e a avaliação da audição devem constituir um processo contínuo ao longo das diferentes etapas do desenvolvimento^(6)^.

A otite média, uma das principais causas de perda auditiva na infância, apresenta incidência global anual de aproximadamente 10,85%, representando mais de 700 milhões de novos casos por ano. Essa taxa pode variar entre regiões e países, sendo de 3,6% na Europa Central para mais de 43% em países da África, em virtude de predisposições genéticas assim como de fatores de risco modificáveis, tais como alergias, infecções de vias aéreas superiores, exposição à fumaça de tabaco, saneamento inadequado, desnutrição e baixo nível socioeconômico^(4)^.

No Brasil, dados da Pesquisa Nacional de Saúde (PNS-2019) apontam que cerca de 1,1% da população com dois anos ou mais relataram “dificuldade extrema ou incapacidade para ouvir”, correspondendo a aproximadamente 2,3 milhões de brasileiros^(7)^. Estudos prévios, realizados com escolares brasileiros, verificaram prevalência de perda auditiva variável entre 5% e 16%, dependendo da faixa etária avaliada e do método de triagem empregado^(8,9)^.

O Brasil dispõe de uma Rede de Atenção à Saúde Auditiva estruturada no âmbito do Sistema Único de Saúde (SUS) desde a Portaria nº 2.073/2004, atualizada e incorporada à Política Nacional de Atenção à Saúde das Pessoas com Deficiência. Essa rede estabelece fluxos integrados para triagem, diagnóstico, concessão de aparelhos auditivos e reabilitação, contando com centros de referência regionais e serviços habilitados em diferentes níveis de complexidade. Embora haja lacunas relacionadas à cobertura, equidade e continuidade do cuidado, o Brasil possui uma das redes públicas de saúde auditiva mais organizadas da América Latina, com expansão progressiva desde a criação do Programa Nacional de Atenção à Saúde Auditiva^(9,10)^.

Já o Programa Saúde na Escola (PSE), instituído pelo Decreto nº 6.286/2007, tem o objetivo de integrar ações de promoção, prevenção e triagem auditiva no ambiente escolar, ampliando o acesso das crianças e fortalecendo a articulação entre os setores de saúde e educação. O PSE tem contribuído para a identificação precoce de alterações auditivas em escolares, favorecendo a continuidade do cuidado em redes municipais de ensino^(11,12)^. Embora o Brasil apresente um modelo de atenção auditiva estruturado, integrado e articulado, com políticas intersetoriais, garantindo acesso a serviços especializados e estratégias de prevenção e triagem em diferentes níveis de atenção à saúde, existem limitações e desigualdades regionais^(11-13)^.

É importante destacar que, na idade escolar, a incidência de perdas auditivas tardias pode ter um aumento de 16,4%, sendo as perdas auditivas de grau leve mais frequentes do que as perdas auditivas moderadas ou profundas^(14-16)^.

A OMS enfatiza que intervenções precoces e de mais baixo custo, como a triagem auditiva, podem reduzir significativamente o impacto social, educacional e econômico da perda auditiva não identificada, especialmente em populações escolares^(4,16)^.

O diagnóstico da perda auditiva é realizado por meio da avaliação audiológica convencional, especialmente pela audiometria de tom puro, que é o padrão-ouro e que necessita de um audiômetro clínico^(17)^. Este fator pode dificultar o acesso à avaliação, já que o audiômetro pode ter custo mais elevado, depende de um profissional habilitado e de local silencioso para a realização^(4,17)^ dificultando o acesso a este serviço em muitas localidades.

Com o avanço da tecnologia, algumas ferramentas têm sido testadas para facilitar a identificação de risco para alterações audiológicas e os resultados obtidos têm favorecido a utilização destes instrumentos em ambientes escolares para ampliação do cuidado à saúde auditiva, mostrando-se válidos para este fim^(18,19)^. No entanto, o ruído presente nestes ambientes pode prejudicar a realização de triagens auditivas, fornecendo resultados falsos-positivo. Para minimizar o impacto do ruído nas avaliações em ambientes sem tratamento acústico, estudo prévio recomendou o uso de fones de ouvido com redução passiva de ruído^(20)^.

Desta forma, diante da dificuldade encontrada quanto à escassez de recursos para a identificação precoce das alterações auditivas na rede pública de saúde, especialmente em países de baixa e média renda, este estudo visa contribuir para o aprimoramento de novos métodos de triagem auditiva automatizada realizada em ambiente escolar, de mais baixo custo, incluindo-a como parte do PSE, em um município do Estado de São Paulo.

Sendo assim, o objetivo do presente estudo foi avaliar o desempenho do aplicativo PETIT (triagem automatizada em tablet), em comparação com a varredura audiométrica, na identificação de risco para alterações auditivas em escolares, utilizando fones de ouvido com cancelamento de ruído passivo.

## MÉTODO

Este trabalho foi aprovado pela Comissão de Ética da instituição, sob o número CAEE 07377319.8.0000.0065. Todos os responsáveis pelas crianças participantes do presente estudo assinaram o Termo de Consentimento Livre e Esclarecido, e as crianças assinaram o Termo de Assentimento.

### Casuística

A amostra deste estudo foi constituída por amostragem não probabilística por conveniência, composta por crianças matriculadas em escolas municipais, selecionadas de acordo com a disponibilidade para participação no período de coleta de dados e conforme os critérios de inclusão. A opção pela amostragem por conveniência justifica-se pelo contexto assistencial em que a pesquisa foi realizada e pela viabilidade de acesso aos participantes no ambiente escolar.

Participaram do estudo 122 crianças matriculadas nos 1os e 2os anos do Ensino Fundamental I, das escolas da rede de ensino público municipal da cidade de Santana de Parnaíba. Os critérios para inclusão no estudo foram: faixa etária entre 05 anos e 08 anos e 11 meses; assinatura do Termo de Consentimento Livre e Esclarecido pelos responsáveis e de Assentimento pelas crianças; meatoscopia sem indicação de presença de corpo estranho e/ou excesso de cerúmen; compreensão, por parte da criança, das instruções para realização da triagem por tablet, varredura e audiometria.

### Materiais e equipamentos

Foram utilizados: Tablet iPad Apple com os aplicativos PETIT^(11)^ e NoiSee; fones HD 280 PRO conectados ao iPad; audiômetro MAICO MA42; fones TDH39; otoscópio Heine mini 3000; e um computador para registro dos dados.

### Procedimentos

O estudo foi conduzido em etapas: autorização dos responsáveis, triagem auditiva com o PETIT, avaliação audiológica completa (composta por meatoscopia, audiometria tonal, vocal e imitanciometria) e triagem por varredura, conforme descrito abaixo. As etapas de desenvolvimento do estudo estão representadas no fluxograma (Figura 1).

**Figura 1:** Fluxograma dos procedimentos do estudo (Santana de Parnaíba, SP, 2025)

#### Triagem auditiva - PETIT

Os sujeitos realizaram a triagem auditiva na própria escola, durante período letivo. Inicialmente foi realizada a inspeção visual do meato acústico e, em seguida, a triagem auditiva.

A triagem auditiva foi realizada com o fone de ouvido HD 280 PRO, por meio do aplicativo instalado em tablet: Programa de Extensão de Triagem Auditiva por Tom – PETIT^(18)^, devidamente calibrado. A triagem ocorre por meio de um jogo interativo com tons puros (warble), utilizando uma combinação de estímulos visuais e auditivos, com paradigma de resposta de escolha forçada (sim ou não), testada e validada em escolares com fone TDH-49^(18)^. O aplicativo utiliza um algoritmo que avalia as frequências de 1000, 2000 e 4000Hz em 20 dBNA e 500Hz em 30 dBNA, em cada orelha separadamente. Para que o indivíduo passe na triagem, são necessárias respostas a no mínimo dois dos três tons emitidos^(21,22)^ 20 dBNA para as frequências de 1000, 2000 e 4000Hz e a 30 dBNA para 500 Hz, em ambas as orelhas. Ao final da triagem, o próprio aplicativo fornece o resultado da triagem como “Passou” ou “Falhou” e o resultado é armazenado no tablet. Na presença de internet via wi-fi, os resultados são enviados para o banco de dados central para posterior análise^(18)^. Durante a análise, considerou-se os resultados incluindo e excluindo a frequência de 500 Hz (por meio de um filtro para análise, que faz parte do aplicativo). Esta análise foi realizada como controle metodológico, devido à maior suscetibilidade de falhas nesta frequência por interferência do ruído presente em ambientes escolares^(18)^.

#### Avaliação audiológica completa

Após as triagens, todas as crianças foram encaminhadas para realização de uma avaliação audiológica completa no centro de referência em Audiologia mais próximo, em Santana de Parnaíba, por fonoaudiólogas deste serviço. Entre os 88 (66,7%) participantes que agendaram a avaliação audiológica completa, 45 (51,1%) compareceram ao serviço especializado e 43 (48,9%) não compareceram. Dos 45 exames realizados, 41 (91,1%) foram recebidos pela pesquisadora, porém apenas 39 estavam completos, contendo todos os registros exigidos para análise (limiares auditivos em todas as frequências testadas, curvas timpanométricas e reflexos acústicos). Desta forma, em virtude da baixa adesão para a realização da avaliação audiológica completa, foi adotada uma nova estratégia para a comparação com os resultados do PETIT, ou seja, a triagem auditiva por varredura.

#### Triagem auditiva - Técnica de varredura

A triagem auditiva por varredura também foi aplicada em ambiente escolar, durante o período letivo, em dia diferente da triagem com o PETIT, utilizando um audiômetro portátil modelo MA42 (MAICO), devidamente calibrado conforme as normas internacionais de segurança, e com o uso de fones de ouvido tipo TDH39.

A triagem auditiva seguiu o protocolo para varredura, com apresentação de tons puros do tipo warble nas frequências de 1000, 2000 e 4000 Hz a 20 dBNA e 500 Hz a 30 dBNA^(21,22)^. O critério de aprovação adotado seguiu diretrizes padronizadas internacionalmente: para ser considerado “Passou” na triagem auditiva, o participante deveria responder corretamente a pelo menos dois dos três tons apresentados nas frequências de 1000, 2000 e 4000 Hz e ao estímulo em 500 Hz, em cada orelha^(21,22)^. Ao final de cada triagem, os resultados foram registrados manualmente pela pesquisadora em protocolo físico padronizado, com a classificação individual de cada participante como “Passou” ou “Falhou”, de acordo com o desempenho observado^(23)^.

### Análise dos dados

Os resultados obtidos na triagem PETIT foram comparados à varredura, para obtenção dos valores diagnósticos da triagem auditiva: sensibilidade, especificidade e acurácia. Para estabelecer a validade da varredura como um procedimento equivalente ao padrão-ouro (audiometria) para as análises, comparou-se a avaliação audiológica completa com a varredura. Foram aplicados modelos de regressão logística binária para avaliar a associação entre os métodos (Modelo 1: Varredura como preditora do resultado no PETIT; Modelo 2: Audiometria convencional como preditora do resultado da varredura). A qualidade do ajuste dos modelos foi analisada com base em: Deviance; AIC (Akaike Information Criterion); BIC (Bayesian Information Criterion); Pseudo-R^2^: McFadden, Cox & Snell, Nagelkerke e Tjur. A significância dos coeficientes dos modelos foi avaliada por meio do Teste de Wald, considerando-se um nível de significância de 5%. As medidas de desempenho diagnóstico — sensibilidade, especificidade e acurácia — foram calculadas com base na matriz de confusão gerada em cada modelo. O teste binomial foi utilizado para comparar as proporções de aprovação observadas na triagem auditiva. Os valores de p, neste caso, referem-se à proporção observada com o valor de referência de 0,5 (50%). As análises foram realizadas utilizando-se o software JASP, versão 0.19.3.0. Os resultados foram considerados estatisticamente significativos quando p < 0,05.

## RESULTADOS

A amostra foi composta por 122 crianças, com idades variando de 5 a 8 anos, sendo a mediana de 7 anos e a média de 6,75 anos (± 0,64). Não houve diferença significante entre os sexos (42,6% sexo feminino; 57,4% sexo masculino; p = 0,123; teste Binomial).

Com o objetivo de verificar a existência de associação entre o desempenho dos participantes no teste PETIT e o sexo, foi aplicado o teste do qui-quadrado de independência com correção de continuidade. Dentre sete indivíduos que falharam na triagem, 3 foram do sexo feminino e 4 do masculino, sem diferença significativa (Qui-quadrado com correção de continuidade: Χ^2^ = 2,608×10^(−30)^; df = 1; p = 1,000).

A Tabela 1 apresenta a análise descritiva da triagem auditiva (por varredura), por frequências testadas em ambas as orelhas, bem como os resultados gerais por orelha e a classificação final de passou (P) ou falhou (F) na varredura. Quando considerados os resultados gerais por orelha, observou-se que 5 participantes falharam em pelo menos uma frequência na OD (4,1%) e 3 na OE (2,5%). Ao final, 6 crianças (4,9%) foram classificadas como tendo falhado na varredura, enquanto 116 (95,1%) passaram. Todas essas proporções foram significativamente diferentes do valor de referência de 50% (p < 0,001), evidenciando um desempenho auditivo geral adequado na amostra avaliada.

**Tabela 1:** Resultados da triagem auditiva por varredura, por frequência e orelha, em escolares da rede pública (Santana de Parnaíba, SP, 2025)

**Variável**	**Resultado**	**Frequência**	**Total**	**Proporção**	**P**
VARREDURA OD 500 Hz	F	1	122	0,008	< ,001
	P	121	122	0,992	< ,001
VARREDURA OD 1000 Hz	F	2	122	0,016	< ,001
	P	120	122	0,984	< ,001
VARREDURA OD 2000 Hz	F	1	122	0,008	< ,001
	P	121	122	0,992	< ,001
VARREDURA OD 4000 Hz	F	3	122	0,025	< ,001
	P	119	122	0,975	< ,001
VARREDURA OE 500 Hz	F	0	122	0,000	< ,001
	P	122	122	1,000	< ,001
VARREDURA OE 1000 Hz	F	3	122	0,025	< ,001
	P	119	122	0,975	< ,001
VARREDURA OE 2000 Hz	F	2	122	0,016	< ,001
	P	120	122	0,984	< ,001
VARREDURA OE 4000 Hz	F	2	122	0,016	< ,001
	P	120	122	0,984	< ,001
VARREDURA OD (geral)	F	5	122	0,041	< ,001
	P	117	122	0,959	< ,001
VARREDURA OE (geral)	F	3	122	0,025	< ,001
	P	119	122	0,975	< ,001
PASSOU/FALHOU VARREDURA	Falhou	116	122	0,951	< ,001
	Passou	6	122	0,049	< ,001

Teste binomial com valor de referência 0,5

**Legenda:** F = falhou; P = passou

Os resultados indicam que, a maioria expressiva das crianças avaliadas foi aprovada no teste PETIT, independentemente da orelha ou condição analisada. Especificamente, 117 crianças (95,9%) foram aprovadas na orelha esquerda, enquanto 116 (95,1%) foram aprovadas na orelha direita. Para verificar uma possível influência do ruído sobre o desempenho dos participantes, foi realizada também a análise se excluindo a frequência de 500Hz. Pode-se observar que as proporções de aprovação permaneceram idênticas para as análises incluindo e excluindo 500 Hz, com 95,9% de aprovação na OE e 95,1% na OD (Tabela 2). Todos os resultados foram estatisticamente significativos (p < 0,001) quando comparados ao valor de referência de 50%, indicando que as taxas de aprovação são significativamente mais elevadas do que o acaso. Ao considerar a classificação geral de aprovação (Passou) ou reprovação (Falhou) no PETIT, observou-se que 115 crianças (94,3%) passaram na triagem auditiva, enquanto 7 (5,7%) falharam.

**Tabela 2:** Resultados da triagem auditiva automatizada com o aplicativo PETIT, com e sem a frequência de 500 Hz, em escolares da rede pública (Santana de Parnaíba, SP, 2025)

**Variável**	**Resultado**	**Frequência (n)**	**Total**	**Proporção**	**p**
PETIT OE	P	117	122	0,959	< ,001
	F	5	122	0,041	< ,001
PETIT OD	P	116	122	0,951	< ,001
	F	6	122	0,049	< ,001
PETIT OE (-500 Hz)	P	117	122	0,959	< ,001
	F	5	122	0,041	< ,001
PETIT OD (-500 Hz)	P	116	122	0,951	< ,001
	F	6	122	0,049	< ,001
PASSOU/FALHOU PETIT	Passou	115	122	0,943	< ,001
	Falhou	7	122	0,057	< ,001

Os valores de *p* referem-se ao teste binomial, comparando a proporção observada com o valor de referência de 0,5

**Legenda:** OE = orelha esquerda; OD = orelha direita; P = passou; F = falhou

Para investigar a relação entre o desempenho dos participantes no PETIT e os resultados da varredura, foi realizada uma análise de regressão logística binária. O objetivo foi verificar se o resultado da varredura (variável preditora) poderia explicar significativamente os escores obtidos no PETIT (variável dependente). A Tabela 3 apresenta os indicadores de ajuste dos dois modelos testados: o modelo nulo (M_0_), que não inclui variáveis preditoras, e o modelo completo (M_1_), que inclui o resultado da varredura como preditor. O modelo M_1_ apresentou um valor de qui-quadrado significativamente menor em comparação ao modelo nulo (Χ^2^(120) = 42,105; p < 0,001), indicando que a inclusão da variável resultado da varredura melhorou substancialmente a capacidade preditiva do modelo. O valor de R^2^ de McFadden foi de 0,785, considerado elevado para modelos logísticos, o que reforça a qualidade do ajuste e o poder explicativo da variável preditora sobre os escores no PETIT. Este valor indica que o modelo explica aproximadamente 78,5% da variância dos resultados do PETIT, o que é altamente satisfatório para este tipo de análise. Os coeficientes estimados e os respectivos testes de Wald estão também apresentados na Tabela 3. Embora o intercepto do modelo M_1_ tenha sido estatisticamente significativo (p < 0,001), o coeficiente associado à variável PASSOU/FALHOU na varredura apresentou resultado estatisticamente não significativo (p = 0,996), com um erro padrão extremamente elevado. Este padrão pode indicar colinearidade ou um ajuste quase perfeito entre as variáveis, o que tende a inflar os erros padrão em modelos logísticos.

**Tabela 3:** Modelo de regressão logística binária para análise do desempenho do PETIT em relação à triagem por varredura (Santana de Parnaíba, SP, 2025)

**Modelo**	**Deviance**	**AIC**	**BIC**	**df**	**ΔΧ^2^**	**p**		**R^2^ McFadden**	**R^2^ Nagelkerke**	**R^2^ Tjur**	**R^2^ Cox & Snell**	
M_0_	53,604	55,604	58,408	121	—	—		—	—	—	—	
M_1_	11,499	15,499	21,107	120	42,105	< ,001		0,785	0,821	0,850	0,292	
**Coeficientes do modelo M_1_ da regressão logística**												
**Variável**			**Estimativa**		**Erro padrão**		**Z**		**Estatística de Wald**	**df**		**p**
Intercepto (M_1_)			−4,745		1,004		−4,724		22,320	1		< ,001
PASSOU/FALHOU VARREDURA			24,311		4390,308		0,006		3,066 × 10^(−5)^	1		0,996

**Legenda:** M_1_ inclui PASSOU/FALHOU VARREDURA

As métricas de desempenho (Tabela 4) indicam uma acurácia total de 99,2%, evidenciando a alta capacidade do modelo em classificar corretamente os casos com base no resultado da varredura. A sensibilidade foi de 85,7%, representando a proporção de casos com alteração auditiva (falha) que foram corretamente identificados pelo modelo. Já a especificidade foi de 100%, indicando que todos os casos com audição dentro da normalidade foram corretamente classificados como tais.

**Tabela 4:** Desempenho do PETIT em comparação à triagem por varredura (Santana de Parnaíba, SP, 2025)

**Observado / Previsto**	**Passou**	**Falhou**	**% de acertos**
Passou	115	0	100,0%
Falhou	1	6	85,7%
**Total geral**			**99,2%**
**Métrica**			**Valor**
Acurácia			0,992
Sensibilidade			0,857
Especificidade			1,000

Com o objetivo de verificar a validade da Varredura como técnica substitutiva à Audiometria Tonal Limiar (considerada padrão-ouro), foi conduzida uma regressão logística binária utilizando o resultado da Varredura como variável dependente, e o resultado da Audiometria como variável preditora. Essa análise foi realizada em caráter complementar, com a finalidade de validar o uso da varredura como técnica comparativa no presente estudo. Assim, esta análise serve como respaldo metodológico, reforçando a legitimidade do uso da varredura nos testes comparativos realizados. A regressão logística aplicada revelou que o modelo com preditor (M_1_) apresentou desempenho superior ao modelo nulo (M_0_), com redução nos valores de Deviance, AIC e BIC, conforme demonstrado na Tabela 5A. A diferença de qui-quadrado entre os modelos foi de ΔΧ^2^(1) = 3,810, com valor de p = 0,051, indicou tendência à significância estatística. Embora o valor de p esteja pouco acima do nível de significância adotado (0,05), os coeficientes de pseudo-R^2^, especialmente o de McFadden (0,241), indicam um ajuste satisfatório do modelo, sugerindo que a varredura está significativamente associada ao resultado da audiometria. A Tabela 5A apresenta também os coeficientes estimados da regressão.

**Tabela 5:** Modelo de regressão logística binária para análise desempenho da triagem por varredura em relação à audiometria tonal (padrão-ouro) (A). Desempenho da triagem auditiva por varredura em comparação à audiometria tonal (padrão-ouro) (B) (Santana de Parnaíba, SP, 2025)

**A – Modelos de regressão logística**														
**Modelo**	**Deviance**		**AIC**	**BIC**		**Df**	**ΔΧ^2^**	***p***		**R^2^ McFadden**	**R^2^ Nagelkerke**		**R^2^ Tjur**	**R^2^ Cox & Snell**
M_0_	15,777		17,777	19,441		38	—	—		—	—		—	—
M_1_	11,967		15,967	19,294		37	3,810	0,051		0,241	0,280		0,224	0,093
**Coeficientes do modelo de regressão logística**														
**Modelo**		**Termo**			**Estimativa (β)**			**Erro Padrão**		***Z***	**χ^2^ de Wald**		***df***	***p***
M_0_		Intercepto			-2,918			0,726		-4,019	16,155		1	<0,001
M_1_		Intercepto			0,000			1,414		≈0	≈0		1	1,000
M_1_		Audiometria (0)			-3,584			1,740		-2,059	4,241		1	0,039
**B - Desempenho**														
**Observado/Previsto**									**Passou (0)**			**Falhou (1)**		**% Correto**
Normal									36			1		97,3%
Alterado									1			1		50,0%
**Total**														**94,9%**
**Métrica**														**Valor**
Acurácia														0,949
Sensibilidade														0,500
Especificidade														0,973

O modelo M_1_ inclui o resultado da Audiometria como variável preditora. A categoria “1” representa falha (alteração). A variável “Audiometria” foi codificada com “1” para falha e “0” para normalidade

O coeficiente da variável preditora (Audiometria) foi estatisticamente significativo (p = 0,039), indicando que o resultado da audiometria é um fator preditivo relevante para o desempenho da criança na varredura. A razão de chances (odds ratio), derivada da exponenciação do coeficiente, indicaria uma queda acentuada nas chances de passar na varredura quando há falha na audiometria — o que reforça a validade da varredura como método substitutivo da audiometria. A Tabela 5B apresenta o desempenho do modelo, que apresentou acurácia geral de 94,9%, com apenas dois erros de classificação (um falso positivo e um falso negativo). A especificidade foi elevada (97,3%), indicando alta capacidade do modelo em reconhecer corretamente os casos normais. Por outro lado, a sensibilidade foi de 50%, refletindo uma capacidade moderada de detecção de casos alterados. Por fim, os valores de pseudo-R^2^ obtidos (McFadden = 0,241; Nagelkerke = 0,280; Tjur = 0,224) estão dentro dos parâmetros esperados para modelos logísticos bem ajustados, o que corrobora a qualidade do ajuste e a utilidade da varredura neste contexto.

## DISCUSSÃO

Em relação ao desempenho auditivo e o sexo dos participantes, os resultados obtidos na triagem PETIT indicam que não houve diferença significativa entre os sexos, corroborando a ideia de que o desempenho auditivo das crianças não foi influenciado por esta variável. Este achado é consistente com estudos anteriores, que não observaram diferença no desempenho entre meninos e meninas em avaliações auditivas similares^(19,24,25)^. Tal evidência sugere que, ao considerar a triagem auditiva, não há necessidade de ajustes ou diferentes abordagens com base no sexo, o que facilita a aplicação universal destes procedimentos.

Em relação a triagem auditiva por varredura, os dados demonstraram que a maioria dos participantes apresentaram função auditiva dentro dos padrões esperados para a faixa etária avaliada, com taxas reduzidas de falha. A taxa de aprovação geral no procedimento foi de 95,1%, sendo que apenas 06 participantes (4,9%) foram classificados como tendo falhado na varredura em pelo menos uma das frequências testadas.

Estudos prévios demonstram variabilidade em relação à taxa de classificação “passa/falha” em triagens auditivas infantis, a depender de fatores como a idade da criança, o ambiente de aplicação, os critérios adotados para reprovação e a metodologia empregada^(25-27)^. Em ambientes escolares, por exemplo, é comum observar uma taxa de falha maior, em razão das condições acústicas menos controladas, da menor colaboração de alguns participantes ou da presença de fatores transitórios, como cerúmen ou infecções de vias aéreas superiores^(14,15,28,29)^.

A heterogeneidade nas taxas de falha em triagens auditivas escolares pode ser atribuída a diversos fatores, incluindo diferenças nos protocolos de triagem, nos critérios de referência, nas condições ambientais durante os testes e nas características demográficas das populações avaliadas. Estudos realizados em diferentes contextos geográficos e socioeconômicos demonstram variações significativas nas taxas de falha, mesmo quando métodos semelhantes, como a audiometria tonal liminar, são empregados^(30).^ Um estudo realizado na Polônia com 67.416 crianças em idade escolar relatou uma taxa de falha de 16,4% na triagem auditiva^(28)^ entre crianças de 6 a 13 anos. Já num estudo realizado na Arábia Saudita^(31)^, com 308 crianças do primeiro ano, foi observada uma taxa de falha na triagem de 26,3% obtida via audiometria tonal. Assim, a taxa de reprovação de 4,9%, observada no presente estudo, se encontra abaixo das observadas nos estudos prévios, podendo ser reflexo das diferenças metodológicas supramencionadas.

Apenas sete participantes (5,7%) falharam no PETIT, indicando uma baixa taxa de falha na triagem. Mesmo com esta baixa prevalência de falhas observada no estudo, ressalta-se a importância da implementação sistemática de protocolos como o PETIT em programas de triagem auditiva infantil, já que os casos identificados como suspeitos de perda auditiva devem ser encaminhados para avaliação audiológica completa, conforme recomendado por diretrizes nacionais^(32)^ e internacionais^(5)^, em virtude da importância do diagnóstico precoce de alterações auditivas, para que não haja prejuízo ao desenvolvimento infantil ou para que este seja mitigado.

Em relação à validade preditiva do PETIT comparado à varredura, os achados demonstraram alta concordância entre os instrumentos, já que indicaram que os resultados da varredura são fortemente associados aos resultados do PETIT, com aproximadamente 80% da variância nos resultados do PETIT sendo explicada pela varredura, sugerindo que o PETIT pode ser considerado um instrumento válido para triagem auditiva infantil.

Do ponto de vista do desempenho, verificou-se acurácia do PETIT com fone HD 280 de 99,2%, especificidade de 100% e sensibilidade de 85,7%. Isso significa que o PETIT com fone com supressão passiva de ruído foi capaz de identificar corretamente todos os indivíduos que passaram na varredura (verdadeiros negativo), bem como a maioria (~86%) dos casos que falharam (verdadeiros positivo), apresentando uma alta acurácia, ou seja, houve a correta classificação de casos “normais” e “alterados” para a grande maioria dos avaliados (99,2%). Estudo anterior^(18)^ identificou que as métricas de desempenho variaram entre os diferentes métodos de triagem auditiva avaliados: as sensibilidades variaram de 60% a 95%, as especificidades de 44% a 91% os valores de acurácia de 49% a 88%; os melhores resultados obtidos foram verificados no método de triagem baseado em tablet e na abordagem em série. Outro estudo^(33)^ com escolares da mesma faixa etária do presente estudo e que utilizou uma triagem auditiva automatizada com fones TDH, em comparação com a audiometria (padrão-ouro) observou acurácia de 85%, sensibilidade de 96% e especificidade de 81%. Sendo assim, o desempenho obtido para o PETIT com fone com supressão passiva de ruído no presente estudo foi semelhante ao obtido em estudos pregressos^(18,33)^.

A validação de instrumentos de triagem é etapa fundamental para assegurar sua aplicabilidade em contextos clínicos e educacionais. O PETIT, por ser de fácil aplicação e de natureza comportamental, mostra-se como uma alternativa viável para contextos com limitações de recursos tecnológicos ou humanos, como escolas e unidades básicas de saúde, conforme estudos prévios^(18,19)^. Os resultados verificados no presente estudo, porém com fones HD 280 PRO, enquanto nos estudos prévios foram utilizados os fones TDH39, sugerem confiabilidade na triagem auditiva realizada por meio do PETIT, indicando que esta pode ser uma ferramenta útil na identificação precoce de risco para alterações auditivas, podendo minimizar os impactos negativos sobre o desenvolvimento da linguagem e o desempenho escolar de crianças, conforme sugerem estudos contemporâneos^(4,25)^.

Para validarmos o uso da varredura como técnica equivalente ao padrão-ouro, fizemos a comparação dos resultados da varredura com a audiometria convencional, para aquelas crianças que realizaram a audiometria e que a avaliação completa foi recebida (n=39). O modelo para esta análise apresentou acurácia de 95%, especificidade de 97% e sensibilidade de 50%. É importante considerarmos, especialmente em razão da sensibilidade observada, que pouco mais de 30% das crianças que entraram nas análises anteriores (PETIT e varredura) realizaram também a audiometria convencional. Sendo assim, possivelmente esta baixa sensibilidade tem relação com a baixa adesão à audiometria e com a baixa prevalência de alterações auditivas detectadas nesta amostra; apenas dois casos que tiveram alteração na varredura fizeram a audiometria e num deles, não houve correspondência entre os resultados. Esse viés amostral é uma limitação reconhecida em estudos com distribuição assimétrica de eventos, impactando diretamente a precisão de medidas como a sensibilidade e os coeficientes logísticos^(25,30)^. Além disso, a sensibilidade pode ser impactada por fatores como critérios de corte e condições ambientais^(25,30)^.

Já a alta especificidade verificada nesta análise é uma característica desejável para testes de triagem populacional, pois minimiza falsos positivos, otimizando o encaminhamento apenas dos casos suspeitos para avaliação diagnóstica completa, evitando a sobrecarga dos serviços especializados^(4,25,30)^. No entanto, quando consideramos a acurácia de 95% entre varredura e audiometria, podemos sugerir que a varredura se apresenta como ferramenta viável para este fim.

Neste contexto e alinhadas às recomendações internacionais^(4)^, estratégias de triagem auditiva que combinem acurácia diagnóstica com facilidade de aplicação e baixo custo são fundamentais para enfrentar a alta prevalência de perdas auditivas, especialmente em populações vulneráveis e em países de baixa e média renda, onde a cobertura de serviços audiológicos especializados é limitada^(34-36)^. A revisão sistemática conduzida por Melo et al.^(30)^ reforça que métodos acessíveis e com bom desempenho diagnóstico são fundamentais para a detecção precoce de perdas auditivas.

Embora não seja objeto do presente estudo, é importante destacar que outras variáveis devem ser consideradas durante programas de triagem auditiva em escolares, tais como o contato prévio com a escola, a realização de anamnese prévia com familiares e professores para obtenção de dados sobre o desenvolvimento e aprendizagem, registro de fatores ambientais e comportamentais que possam interferir nos resultados, entre outras^(1-4,37,38)^.

Ao discutirmos o desempenho da triagem auditiva em escolares, é fundamental analisar também o custo-benefício do programa, incluindo fatores logísticos e humanos envolvidos no processo. Em um relatório sobre triagem auditiva da OMS, foi apresentada uma avaliação dos custos e retornos financeiros associados à implementação de estratégias de triagem auditiva e de serviços de intervenção precoce em diferentes contextos. Segundo o documento, a expansão global da cobertura de triagem auditiva e de programas de intervenção nos próximos dez anos demandaria um investimento adicional anual per capita de aproximadamente US$ 1,33, resultando em ganhos expressivos em saúde pública, com a prevenção estimada de cerca de 130 milhões de anos de vida ajustados por incapacidade, benefício direto a 1,4 bilhão de indivíduos e retorno econômico médio de US$ 16 para cada dólar investido. Tais dados evidenciam a relevância da triagem auditiva, ressaltando a necessidade da ampliação e fortalecimento dentro dos programas nacionais de saúde auditiva infantil e escolar^(37)^.

No contexto brasileiro, a análise do custo-benefício das triagens auditivas deve considerar, também, as barreiras estruturais que podem comprometer a continuidade do cuidado, como o tempo de espera para avaliação diagnóstica, a escassez de profissionais especializados em determinadas regiões e a necessidade de deslocamento das famílias até os serviços de referência. Um estudo de abrangência nacional verificou que o tempo médio de espera para atendimento especializado em serviços de referência em saúde auditiva pode ultrapassar seis meses, especialmente em municípios que não dispõem de centros habilitados pelo SUS^(12)^.

Os resultados do presente estudo corroboram e ampliam os achados de investigação prévia^(3,9)^, que avaliou o desempenho do PETIT com o fone HD 280 PRO em condições controladas de escuta, utilizando ruído branco e ruído de cafeteria como simulações de ambientes ruidosos, em adultos. Naquele estudo, os autores observaram sensibilidade e especificidade de 100% quando o HD 280 PRO foi utilizado em conjunto com o PETIT, alcançando acurácia de 100% em ambos os cenários de ruído avaliados e desempenho superior ao do fone TDH. No presente estudo, a mesma ferramenta foi aplicada em ambiente real e na população-alvo, obtendo-se acurácia de 99,2%. Esse achado reforça a hipótese de que o HD 280 PRO constitui alternativa tecnicamente viável ao fone TDH-39, tradicionalmente empregado em audiometrias clínicas, especialmente em contextos nos quais a triagem auditiva é realizada em ambientes não controlados, como escolas. Mesmo diante das potenciais fontes de ruído ambiental típicas do ambiente escolar, o HD 280 PRO apresentou desempenho equivalente ao observado em laboratório. A comparação entre os estudos permite sugerir que o desempenho diagnóstico verificado no presente trabalho é compatível com o relatado por Aquino^(38)^.

Adicionalmente, destaca-se que o fone HD 280 PRO apresenta custo inferior ao do fone TDH-39 e dispõe de tecnologia de atenuação passiva de ruído, o que o torna particularmente adequado para aplicações em ambientes escolares, nos quais o controle acústico é limitado. O custo estimado do HD 280 PRO é de aproximadamente US$ 89^(39)^, enquanto o do TDH-39 é de cerca de US$ 500^(40)^.

Sendo assim, nossos resultados sugerem que o protocolo ora avaliado se apresenta como uma ferramenta viável para identificar precocemente alterações auditivas em escolares, consistente com a literatura nacional e internacional, que valida o uso de instrumentos como o PETIT em contextos escolares e clínicos por sua sensibilidade à detecção de alterações auditivas mínimas^(2,11,12,18)^.

A adoção do fone de ouvido com cancelamento passivo de ruído em conjunto com o PETIT revelou-se vantajosa, tanto pela facilidade de manuseio quanto pelo baixo custo envolvido, o que amplia suas possibilidades de uso em regiões remotas, vulneráveis ou com poucos recursos técnicos e humanos disponíveis. Embora o fone TDH-39 seja amplamente utilizado em triagens auditivas, seus custos e limitações logísticas podem inviabilizar sua aplicação em grande escala no contexto educacional. Dessa forma, o PETIT com fones de mais baixo custo surge como uma alternativa viável, com potencial para integrar políticas públicas voltadas à promoção da saúde auditiva.

Apesar dos resultados promissores, algumas lacunas permanecem e demandam investigação futura. Primeiramente, é necessário expandir a diversidade amostral, incluindo diferentes faixas etárias, regiões geográficas e contextos escolares, a fim de possibilitar a generalização dos achados. Além disso, destaca-se a necessidade de novos estudos que investiguem o uso de dispositivos móveis na triagem auditiva, bem como estudos sobre a análise de custo-benefício da implementação de instrumentos de baixo custo para triagem auditiva, no contexto escolar.

## CONCLUSÃO

Nossos resultados indicaram que a triagem auditiva via tablet com o uso de fones com cancelamento passivo de ruído demonstrou valores de acurácia de 99,2%, sensibilidade de 85,7% e especificidade de 100%, o que reforça sua validade como instrumento de triagem. Assim, a triagem auditiva realizada com fones de ouvido de mais baixo custo mostrou-se como ferramenta promissora para ambientes escolares, especialmente em contextos com infraestrutura limitada.
